# Unbalanced predatory communities and a lack of microbial degraders characterize the microbiota of a highly sewage-polluted Eastern-Mediterranean stream

**DOI:** 10.1093/femsec/fiae069

**Published:** 2024-04-29

**Authors:** Yossi Cohen, Julia Johnke, Alfred Abed-Rabbo, Zohar Pasternak, Antonis Chatzinotas, Edouard Jurkevitch

**Affiliations:** Department of Plant Pathology and Microbiology, Institute of Environmental Sciences, Faculty of Agriculture, Food and Environment, The Hebrew University of Jerusalem, Rehovot, 76100, Israel; Presently at DayTwo, Rehovot, Israel; Evolutionary Ecology and Genetics, Zoological Institute, University of Kiel, Kiel, Germany; Faculty of Science, Bethlehem University, Palestine; Department of Plant Pathology and Microbiology, Institute of Environmental Sciences, Faculty of Agriculture, Food and Environment, The Hebrew University of Jerusalem, Rehovot, 76100, Israel; Presently at the Division of Identification and Forensic Science, Israel Police, National Headquarters; Department of Applied Microbial Ecology, Helmholtz Centre for Environmental Research - UFZ, Permoserstrasse 15, 04318 Leipzig, Germany; Institute of Biology, Leipzig University, Talstrasse 33, 04103 Leipzig, Germany; Centre for Integrative Biodiversity Research (iDiv) Halle-Jena-Leipzig, Puschstrasse 4, 04103 Leipzig, Germany; Department of Plant Pathology and Microbiology, Institute of Environmental Sciences, Faculty of Agriculture, Food and Environment, The Hebrew University of Jerusalem, Rehovot, 76100, Israel

**Keywords:** *Bdellovibrio* and like organisms, predation, protists, river, sewage, water pollution

## Abstract

Wastewater pollution of water resources takes a heavy toll on humans and on the environment. In highly polluted water bodies, self-purification is impaired, as the capacity of the riverine microbes to regenerate the ecosystem is overwhelmed. To date, information on the composition, dynamics and functions of the microbial communities in highly sewage-impacted rivers is limited, in particular in arid and semi-arid environments. In this year-long study of the highly sewage-impacted Al-Nar/Kidron stream in the Barr al-Khalil/Judean Desert east of Jerusalem, we show, using 16S and 18S rRNA gene-based community analysis and targeted qPCR, that both the bacterial and micro-eukaryotic communities, while abundant, exhibited low stability and diversity. Hydrolyzers of organics compounds, as well as nitrogen and phosphorus recyclers were lacking, pointing at reduced potential for regeneration. Furthermore, facultative bacterial predators were almost absent, and the obligate predators *Bdellovibrio* and like organisms were found at very low abundance. Finally, the micro-eukaryotic predatory community differed from those of other freshwater environments. The lack of essential biochemical functions may explain the stream's inability to self-purify, while the very low levels of bacterial predators and the disturbed assemblages of micro-eukaryote predators present in Al-Nar/Kidron may contribute to community instability and disfunction.

## Teaser

This study shows that the microbes of a severely sewage-impacted intermittent stream lack the biochemical and ecological potential needed to purify it and maintain the ecosystem's stability.

## Introduction

In many parts of the world, sewage flows to nearby rivers without treatment, transforming an essential life-sustaining resource into a cause of harm to health and environment. Under high inputs of sewage containing high loads of pathogens, high concentrations of organic matter, chemicals and mineral nutrients, water quality deteriorates as the capacity of the riverine microbes to regenerate the ecosystem is overwhelmed (Pascual-Benito et al. 2020, Reddy and Dubey 2021). While recent studies show that sewage drastically alters the aquatic microbiome's composition and dynamics (Korajkic et al. 2015, Zhang et al. 2015), information on the composition, dynamics and functions of microbial communities in highly sewage-impacted rivers, which are common in many parts of the world, is restricted. In that sense, wastewater treatment plants (WWTPs) provide a valuable comparison with highly sewage-polluted streams as their raw material is similar. Yet in WWTPs, sewage is efficiently purified to reclaimed water by the plant's microbial communities, which are under ecological selection by the constructed environment and the operational conditions (Wang et al. 2012, Cohen et al. 2019). Depending on the quality of the released reclaimed water into the environment, microbial communities are mostly locally disturbed (Korajkic et al. 2015, Pascual-Benito et al. 2020). Numerous studies have shown that WWTPs are enriched in populations performing tasks such as nitrification, organic material breakdown, phosphate accumulation and much else (Ju and Zhang 2015, Cohen et al. 2019, among others). Also, fecal and pathogenic microorganisms decay, for example, through competition, predation and exposure to unfit conditions (Korajkic et al. 2019).

Predation is thought to play an important role in shaping the dynamics and composition of bacterial communities in different environments (Chauhan 2009; Shapiro et al. 2009, Johnke et al. 2014, Hungate et al. 2021), but large-scale and time-series analyses of microbial predators are rare and almost exclusively focus on micro-eukaryotes (Pauli et al. 2001, Madoni 2011, Zahedi et al. 2019). Lately, the generalization of high-throughput 16S rRNA gene community analysis has uncovered the ubiquitous presence and responsiveness of bacterial predators such as the bacterial predators *Bdellovibrio* and like organisms (BALOs) (Chauhan 2009, Feng et al. 2017, Cohen et al. 2021, Hungate et al. 2021). They appear to form very diverse, common communities able to prey on a very large variety of bacteria (Kandel et al. 2014, Feng et al. 2017, Cohen et al. 2021).

So far, very few studies have examined community composition and dynamics of raw sewage-highly impacted rivers. The aim of this study was to fill that knowledge gap and to address the hypothesis that the self-purification potential of such streams is undermined because biochemical and ecological functions provided by the bacterial and by the micro-eukaryote communities are lacking. In order to achieve this goal, the highly sewage-contaminated Al-Nar/Kidron (ANK) intermittent stream that originates in Jerusalem and flows to the Dead Sea (Fig. S1) was sampled for 1 year. We used rRNA-gene high-throughput sequencing to analyze the microbial community at large, including Bacteria, and more specifically BALO predators, and the micro-eukaryotes, while monitoring the chemical and physical environmental parameters. The results of these analyses were contextualized versus published data from other rivers and from a WWTP located in Al-Bireh (Cohen et al. 2019), distant by ∼25 km, and fed inputs similar to those entering the ANK stream.

## Materials and methods

### Sampling

The ANK river is a seasonal, winter stream with its head in Wadi Joz in Jerusalem (750 m). It flows eastward on a 34 km-long course to reach the shores of the Dead Sea at Avnat (-415 m; refer to Figure S1 for more details, see Supplementary materials and methods). Surface river (0–15 cm) water was collected in 1-L sterile jars in triplicate. The samples were kept on ice until delivery to the laboratory within a few hours, for chemical and physical analysis at Bethlehem University. The rest were refrigerated and transferred on ice to the Hebrew University within 24 h, for DNA extraction and further processing. Sampling was performed once during the first week of the month for 1 year from March 2013 to February 2014 and, additionally, for four consecutive weeks in August 2013 and in February 2014, at sites along a stretch of ∼12 km, in and near Al-Ubeidiya, depending upon accessibilityFigure S1B).

### Chemical and physical analysis of water samples

Water analysis was performed to measure the following parameters: temperature, pH, salinity, conductivity, total organic carbon (TOC), biological oxygen demand (BOD), chemical oxygen demand (COD), NO_3_, NO_2_, NH_4_^+^, PO_4_^−^, total suspended solids (TSS) and total dissolved solids (TDS), as per Cohen et al. (2019, 2021), according to standard methods.

### DNA extraction

Two ml of water of each replicate from each sampling time were centrifuged at 10 000 g for 10 min at 4°C, washed with sterile double distilled water and centrifuged again. The pellet was suspended at 1.2 ml of double distilled water (DDW) and then processed using a PowerSoil^TM^ DNA Extraction Kit (MoBio Laboratories, USA), according to the manufacturer's instructions. DNA concentration and purity were measured using Nanodrop^TM^ 2000 (Thermo Scientific, Wilmington, DE, USA) and was in the range of 20–100 ng.µl^−1^ for all samples.

### Bacterial quantification by real-time qPCR

qPCR was performed as in Cohen et al. (2021). Briefly, standards were prepared by inserting a 1467-bp fragment of the *Bdellovibrio bacteriovorus* HD100 and *Bacteriovorax stolpii* UKi2 strain 16S rRNA gene amplified with primers 27F and 1492R, respectively, into a PGEM-T easy plasmid vector system (Promega, WI, USA). Ten-times serial dilutions from 10^3^ to 10^10^ plasmid copies per reaction were used to construct standard qPCR curves and plasmid copy numbers were calculated. For total bacteria, the primer pair 1048F-1175R was used to quantify the 16S rRNA gene copy number. For additional details, see Supplementary materials and methods.

### 16S rRNA and 18S rRNA gene community sequencing and analysis

This analysis was performed as in Cohen et al. (2019, 2021). MiSeq Illumina sequencing (Carlsbad, CA, USA) was performed using bacterial 16S rRNA primers 515F (5′GTGCCAGCMGCCGCGGTAA-3′) and 806R (5′- GGACTACHVGGGTWTCTAAT-3′) targeting the V4 region, Bdellovibrionales primers Bd824F (‘5-ACTTGTTGTTGGAGGTAT-3′) and Bd1222R (‘5-TTGTAGCACGTGTGTAG-‘3), Bacteriovoracales primers Bx341F (5′-CTACGGGAGGCAGCAG-3′) and Bx672RC (5′-TACCCCTACATGCGAAATTCC-3′), and eukaryotic 18S rRNA primers Euk_1391 (5′-GTACACACCGCCCGTC-3′) and Euk_Br (5′-TGATCCTTCTGCAGGTTCACCTAC-3′) targeting the V9 region. For additional details, see Supplementary materials and methods.

## Results and discussion

Averages of 46 K of and 16.2 K reads per sample were obtained for Bacteria 16S rRNA and Eukarya 18S rRNA gene-targeted sequencing, respectively, enabling almost complete coverage of the communities (Figure S2A-D). The number of Bd and Bx amplicons obtained was lower (7924 and 2920, respectively), yet, for both BALOs, high coverage based on Good's coverage was achieved (Figure S2B-C).

**Figure 1.**
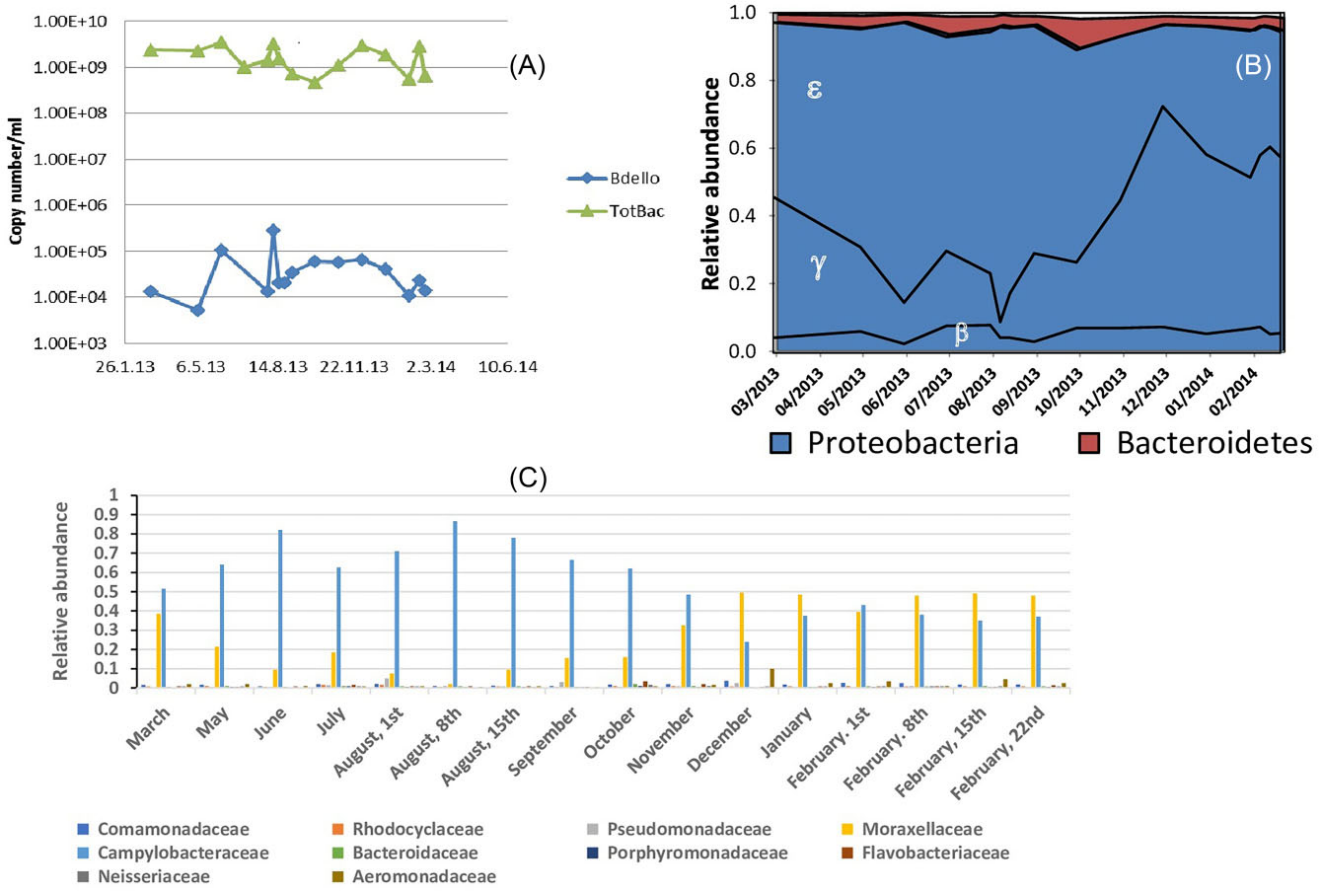
(A) Quantitative PCR of the 16S rRNA gene copy number for Bacteria and *Bdellovibrio* during a year-long sampling effort at the Al Nar/Kidron (ANK) stream. (B) Distribution of taxonomically classified 16S rRNA gene-based OTUs at the family level. (C) Yearly dynamics of the dominant bacterial clades at the ANK stream; the Greek letters refer to classes in the Proteobacteria.

Total bacteria in the ANK stream, as reckoned by 16S rRNA copy numbers, was high (∼2.10^9^ copy/ml) (Fig. 1A), on a par with those measured in the liquor (the effluent-like fraction) of WWTP secondary treatment reactors (Cohen et al. 2019). Diversity of the Bacteria and of the micro-eukaryotes was lower than in other rivers, river systems, microcosms impacted by WWTP-effluent or by urban water (Table 1 and Table S1) (Korajkic et al. 2015, Li et al. 2018, Wang et al. 2018, Cohen et al. 2019, Nakatsu et al. 2019, Pascual-Benito et al. 2020, Xu et al. 2020, Abdullah Al et al. 2021, Muhammad et al. 2021, Shang et al. 2021, Ting et al. 2021). More specifically, the α-diversity estimates of the ANK stream's Bacteria and of the micro-eukaryote communities were significantly different, but their values were much closer than those observed in the nearby Al-Bireh (AB) WWTP (Table 1) (Cohen et al. 2019). Furthermore, Bray–Curtis distances of both microbial communities were significantly lower than those calculated from the AB WWTP data (Cohen et al. 2019) (Bacteria: ANK, 0.372; AB flocs, AB liquor: 0.548, 0.777; micro-eukaryotes: ANK, 0.275; AB flocs, AB liquor: 0.707, 0818, *P* < 0.0001). This can be explained by the different bacterial community structures between ANK and AB, and by the strong seasonal variations observed in the ANK river data (MRPP-A=0.223, *P* < 0.001, Figure S3A, B). Thus, the ANK river's microbial communities exhibited reduced diversity and reduced stability compared with other fresh water bodies in which sewage pollution was less severe.

**Table 1.** α-diversity estimates (S=Richness, E=Evenness, H=Shannon index, D’= Simpson index), and standard error, of bacterial and eukaryotic communities of the Al-Nar/Kidron (ANK) river, calculated based on OTU grouping of 16S rRNA gene and 18S rRNA gene sequences. The Al-Bireh (AB) WWTP data (italics) are from Cohen et al. (2019) and are provided for comparison; F, floc fraction; L, liquor fraction.

	ANK	AB F	AB L
**16S rRNA**			
**S**	**802.1 ± 57.1**	*1494.1 ± 122*	*1223 ± 432.2*
**E**	**0.36 ± 0.1**	*0.65 ± 0.1*	*0.55 ± 0.2*
**H**	**2.46 ± 0.5**	*4.76 ± 0.7*	*3.96 ± 1.5*
**D**	**0.70 ± 0.1**	*0.92 ± 0.1*	*0.84 ± 0.2*
**18SrRNA**			
**S**	**691.1 ± 33.9**	*902.6 ± 74.1*	*688.3 ± 128.6*
**E**	**0.34 ± 0.03**	*0.50 ± 0.1*	*0.33 ± 0.1*
**H**	**2.24 ± 0.3**	*3.4 ± 0.6*	*2.21 ± 0.9*
**D**	**0.73 ± 0.1**	*0.87 ± 0.1*	*0.70 ± 0.2*

More than 90% of the operational taxonomic units (OTUs) detected in the ANK samples were affiliated to the Proteobacteria (Fig. 1B). γ- and ε-proteobacteria largely dominated, comprising ∼90% of the sequences. They were mainly composed of members of the families Moxarellaceae (γ-proteobacteria, mostly *Acinetobacter*) and Campylobacteriaceae (ε-proteobacteria, almost exclusively *Arcobacter*). Moxarellaceae and Campylobacteriaceae reached maxima in winter (relative abundance RA = 0.58), and in summer (RA = ∼0.87), and their lowest levels in summer (RA = ∼0.03 RA, significant difference with the former's summer maximum, Wilcoxon rank-sum, *P* = 0.011) and in winter (RA = 0.23; no significant difference with the latter's summer maximum, Wilcoxon rank-sum, *P* = 0.5238), respectively (Fig. 1C). This pattern was in line with the significant Pearson correlations between bacterial community structure analysis and temperature (Table S2). Other, weaker sources of variation ((|r|) > 0.45) included salinity and nitrate (Table S2). The high TSS and BOD values pointed to the high levels of organic compounds present in the water. They were higher than those measured in the liquor fraction of WWTPs (Cohen et al. 2021), and above the limits of ∼30 mg.l^−1^ set for direct environmental wastewater discharge (UK Environment Agency 2019) (Table S3). The γ- and ε-proteobacteriaare numerically important classes in WWTPs, as are the β- proteobacteria (Ju and Zhang 2015, Saunders et al. 2016, Cohen et al. 2019). The latter are also common in the water column of rivers (Zeglin 2015), but in the ANK stream, they were detected at low levels at all times (0.08 ≥ RA ≥ 0.026). Although Bacteroidetes diversity was consequent (227 OTUs), these bacteria were present at low levels (0.06% ± 0.001%) (Fig. 1C, Table 2). Bacteroidetes are found at high abundances in WWTPs and in the water column of streams, even in pollution/sewage-impacted ones (Zeglin 2015, Jordaan and Bezuidenhout 2016). The large number of peptidases, glycoside hydrolases and glycosyl transferases their genomes encode for (Fernández-Gomez et al. 2013) allow them to grow attached to organic matter particles and hydrolyze them, as well as to degrade dissolved organic material. Here, *Acinetobacter* spp. were present at a high RA. They can degrade aromatic compounds and hydrocarbons, and many strains are lipolytic (Jung et al. 2015); *Aeromonas* spp. were conspicuously present (>1.8% RA) and can degrade chitin (Seshadri et al. 2006). Together, this suggests that some organic compounds can be degraded in ANK water, but these functions do not replace those performed by the missing species (Table 2). Other taxa playing important roles in water purification, such as nitrification, denitrification, hydrolysis and phosphorus accumulation, were also found at low to very low levels in the ANK samples (Table 2) compared with WWTPs (Ju and Zhang 2015, Cohen et al. 2019, Zhang et al. 2019). More specifically, the nitrifiers *Nitrosomonas* and *Nitrospira*, and *Nitrospina* and *Nitrotoga*, were hardly detected or were not found at all, respectively (Table 2). This suggests a low potential for nitrification, which could explain the relatively high ammonium levels (Table S3). In polluted rivers, ammonium concentration was found to have a stronger influence on the composition of microbial communities than did nitrate, the levels of which were also high in the ANK stream (Table S3) (Shang et al. 2021), maybe because denitrifiers were one or more orders of magnitude lower than in WWTPs (Table 2; Cohen et al. 2019).

**Table 2.** Heatmaps of minimum, maximum and averaged relative abundance (RA), standard deviation and occurrence in samples (%) of bacterial taxa found in the Al-Nar/Kidron (ANK) stream, and known to perform specific ecological functions according to Ju and Zhang (2015). RA values of heatmaps and occurrence (%) values are presented in the last row and right most column, respectively. Species in orange-colored cells are missing or found at very low levels; in white cells, they are at levels comparable with WWTPs ( ± 1 order of magnitude) (Cohen et al. 2019); in the light green cells, these are species at higher levels than in the ANK stream.

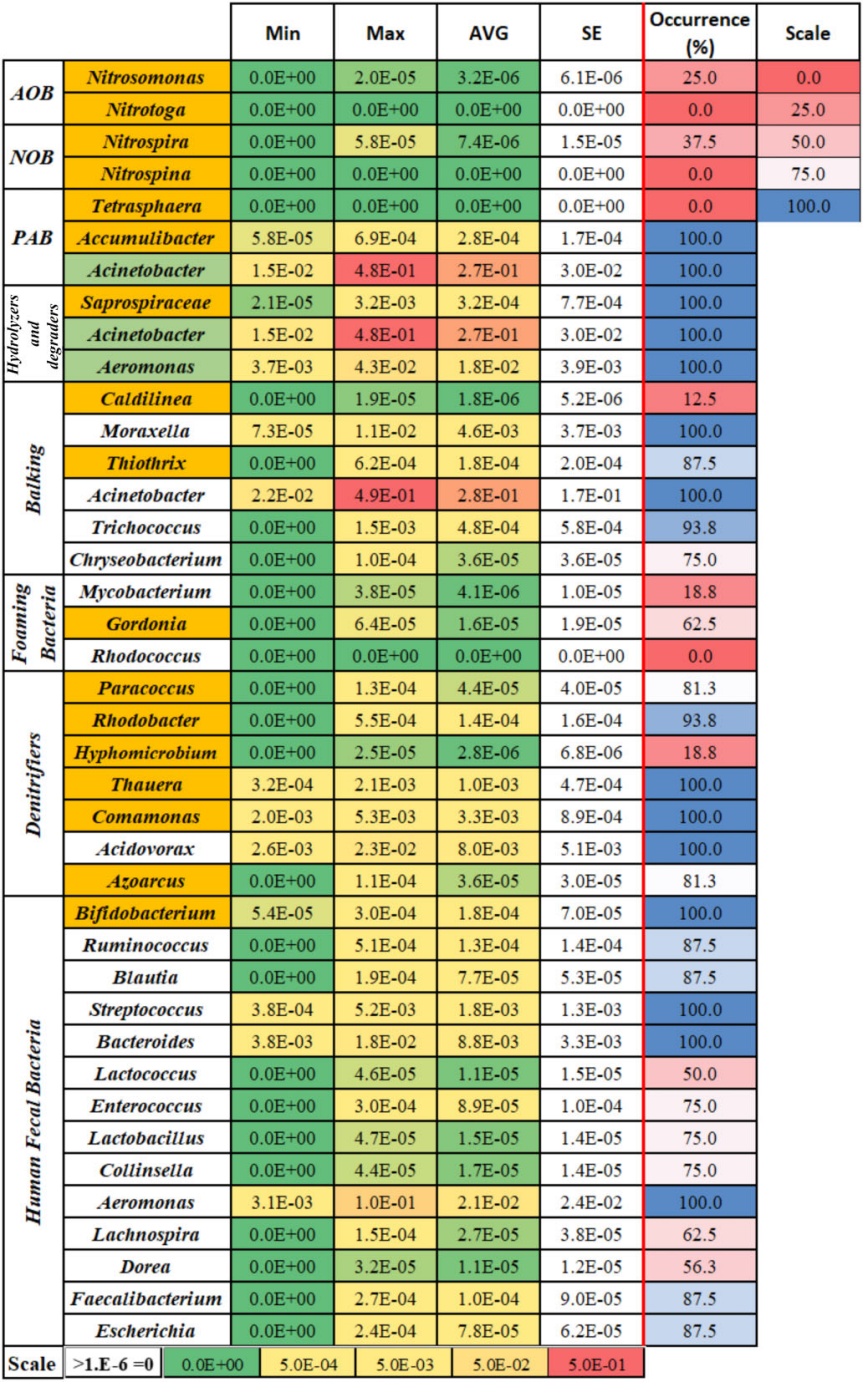

This, despite the high density of *Arcobacter* and *Acinetobacter*, species that include efficient denitrifying strains that can be greatly enriched for in nutrient removal or sludge-based denitrification cultures (Pishgar et al. 2019, Xia et al. 2020), suggests that these were not selected for or not active in the stream. Phosphate accumulators were rare, as *Tetrasphaera* were absent and *Accumulibacter* was very scarce (Table 2). In that sense, the high concentrations of *Acinetobacter* may (partly) fill that role and reduce phosphorus to levels (6.3 ppm) in the range of WWTP effluents (5–20 ppm) (Cloete et al. 1985, Yeoman et al. 1988). Three common clades of complex organic compound degraders in WWTPs were almost completely absent in ANK, despite the high organic loads: Myxococcales (0.2%), which include numerous facultative predators (Shimkets 1990); floc-forming Chloroflexi (0.03%), which feed on lysed bacterial cell debris (Speirs et al. 2019); and Planctomycetes (0.02%), which mainly associate with particles, surfaces and hosts (Wiegand et al. 2018, 2020). Human fecal bacteria, on the other hand, did not appear to be more represented in the ANK water samples than in the AB and other WWTP samples (Cohen et al. 2019). Yet, the very high levels of the opportunistic pathogens *Acinetobacter* and *Aeromonas* should certainly be noted.

**Figure 2.**
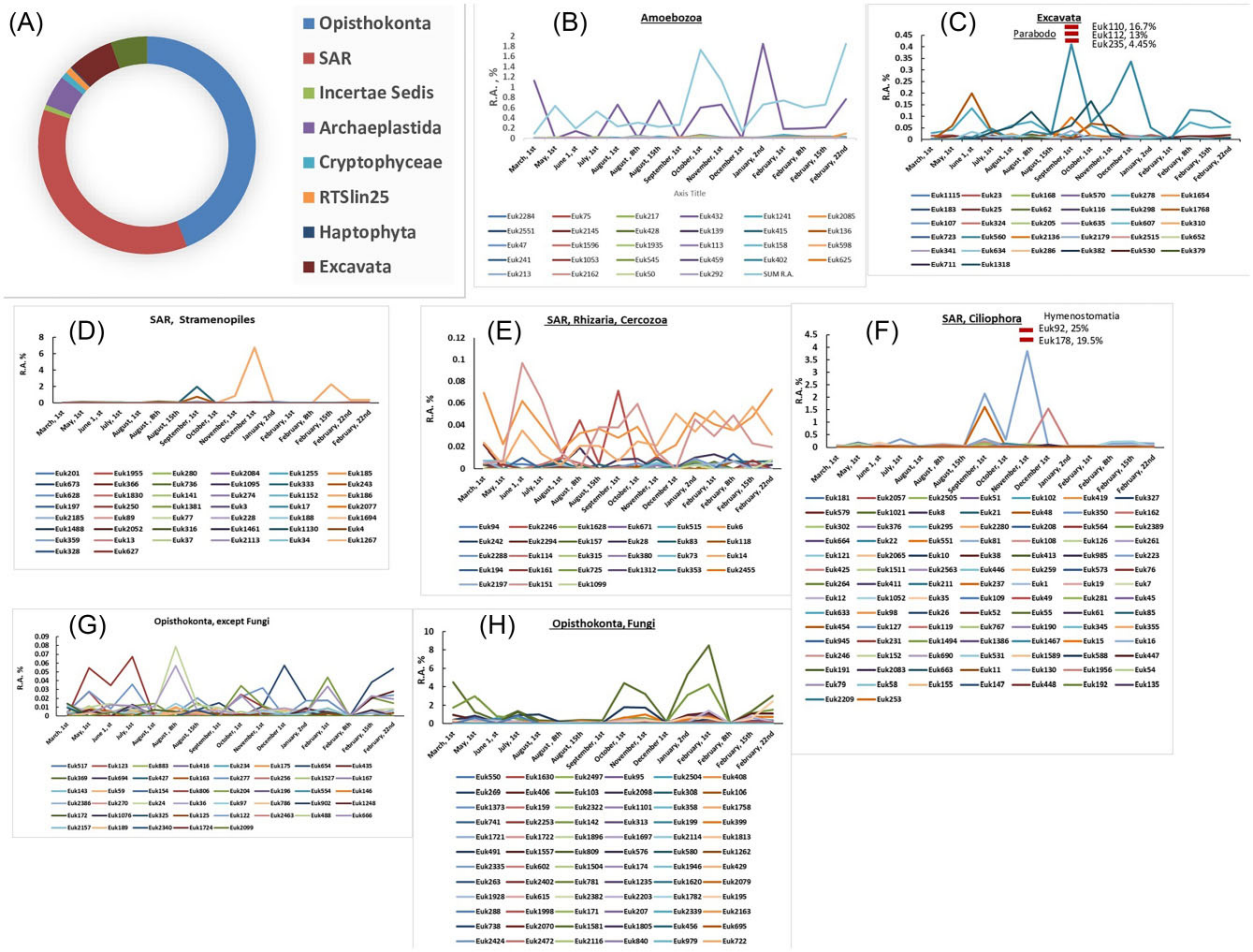
(A) Pie representation of the distribution of identified microeukaryote phyla. (B–H) Dynamics of all OTUs in microeukaryote phyla/classes/orders along the year-long time series in the ANK stream. In Excavata and in the SAR, Ciliophora, highly abundant OTUs at specific time points are shown as short red bars, with relative abundance (RA) in %.

The identified taxa in the micro-eukaryote community analysis accounted for 31.7% of the total OTUs. The Opisthokonta (animalia, fungi) accounted for 43.6% of identified sequences, followed by the Stramenopiles, Alveolates and Rhizaria (SAR) Supergroup (11.4%), and the Archaeplastida (algae, plants, 5%) (Fig. 2A). The most dominant (>1%) OTUs are listed in Table 3, and accounted for 71.3% of total RA, except for the low evenness value (Table 1). Most were unidentified, and the identified taxa were mostly not predatory. Yet, among all sequences, numerous potential bacterial predators were identified (Table S4; in total, 238 OTUs). They were dominated by SAR Ciliophora, Excata and Amoebozoa. Among those, dominant OTUs included three Parabodo (Excavata) and two Hymenostomatia (SAR) that greatly varied throughout the year, peaking at 16.7%, 13% and 4.45% in September, and at 25% and 19.5% in December, respectively (Fig. 2B-H). Arachnids, rotifers and nematodes, which can also consume bacteria and protists, accounted for 4% of the sequences. Remarkably, most OTUs exhibited maximal RA from the end of September to February, except for Rhizaria and some non-fungi Opiskhonta (mostly Metazoa) that fluctuated during the whole year (Fig. 2B-H).

**Table 3.** Relative abundance (RA; annual average and standard error, in %) of the most abundant micro-eukaryote single OTUs above 1% RA. When available, taxonomic affiliation is at the family/genus level, along with a general description of feeding habits.

Phylum	Family/genus	RA (%) ± SE	Details
N/A		11.5 ± 0.8	
N/A		8.6 ± 0.9	
N/A		7.9 ± 0.4	
N/A		6.0 ± 0.4	
N/A		5.6 ± 0.3	
N/A		4.4 ± 0.7	
Archaeplastida	Chlorophyceae/Haematococcus	3.7 ± 0.7	(Pseudo)flagellated green alga, photosynthetic and saprotrophic
N/A		3.4 ± 0.4	
N/A		2.9 ± 0.5	
N/A		2.6 ± 0.2	
Opisthokonta	Ascomycota/Pezizomycotina	2.5 ± 0.5	Yeast, heterotrophs
N/A		2.4 ± 0.3	
N/A		1.9 ± 0.1	
SAR	Hymenostomatia/Agolohymena	1.8 ± 1.1`	Histophagous tetrahymenid ciliate
Excavata	Parabodonida/Parabodo	1.5 ± 0.7	Heterotrophic flagellates, bacterial grazer or animal parasites
Excavata	Parabodonida/Parabodo	1.3 ± 0.6	Heterotrophic flagellates, bacterial grazer or animal parasites
Opisthokonta	Dipodascaceae/Magnusiomyces	1.3 ± 0.2	Yeast, heterotrophs
N/A		1.2 ± 0.1	
N/A		1.1 ± 0.1	
**Total RA**		**71.3**	

The micro-eukaryote community in the ANK stream greatly differed from those found in WWTPs and in rivers. In well-functioning WWTPS, Rhizaria (Cercozoa), Stramenopiles and Peritrich predators dominate (Madoni 2011, Cohen et al. 2019, Zahedi et al. 2019). By contrast, all were low or very low in ANK data, averaging 0.13% ± 0.006%, 1.07% ± 0.008% and 0.42% ± 0.003%, respectively. Moreover, while the diatoms, Chrysophyceae, Dinoflagellates and Ochrophyta (Bacillariophyceae) were very abundant in rivers of water qualities ranging from slightly to heavily polluted (Korajkic et al. 2015, Li et al. 2018, Xu et al. 2020, Abdullah Al et al. 2021, Ingala et al. 2021, Muhammad et al. 2021, Choi et al. 2022), these taxa were almost absent from the ANK river samples.

These analyses of bacterial and micro-eukaryote communities and of ANK river water support the notion that heavy sewage pollution along with extreme climatic conditions (Zituni et al. 2021) resulted in specific assemblages unable to provide water-purifying functions such as clarification, biochemical oxygen demand and turbidity reduction by organic matter (OM) degradation and nitrification, which are known to depend upon balanced predatory activities (Madoni 2011). In turn, the restricted potential for degradation suggests that the ANK river's microbes mostly tolerate but do not degrade the contaminants (Chakraborty and Bhadury 2015).

In order to further address the potential role of bacterial predators in this severely polluted ecosystem, a BALO-targeted 16S rRNA gene community analysis was performed. It revealed that Bbellovibrionaceae (Bd) and Bacteriovoracaceae (Bx) diversities were lower than those observed in WWTPs, respectively (Cohen et al. 2021) (Table 4; t-test, *P* < 0.01, two-tailed). The BALO predators were present at very low relative abundances in the 16S rRNA gene reads of the general Bacteria, averaging 0.004% ± 0.00085% for the Bd and 0.009% ± 0.001% for the Bx. These low levels were confirmed by qPCR targeting the Bd community (Fig. 1). Thus, while the diversity of the BALO community (Bd+Bx) was lower—but not extremely different from that observed in relevant habitats (WWTPs and freshwater) (Paix et al. 2019, Ezzedine et al. 2020, Cohen et al. 2021)—its abundance was greatly reduced. The abundance of Gram-positive taxa (non-prey) or a negative effect of the high organic matter content in the river as possible causes for the low abundance of BALOs cannot be invoked as Gram-positive cells constituted only ∼15% of the bacterial community (Firmicutes and Actinobacteria mostly). Moreover, BALOs are abundant in WWTP sludge in which the content of organic matter is very high (Feng et al. 2017, Cohen et al. 2021). In effect, BALOs appear to be more abundant in organic matter-rich environments, probably because these sustain large bacterial communities that may be preyed upon, such as in WWT schemes or in soils with a high organic matter content (Jurkevitch 2020, Petters et al. 2021). A possible clue to the low BALO abundance in the ANK river may be the high ammonium concentrations (Tables S3 and S5), which were found by Paix et al. (2019) to negatively correlate with Bd populations in peri-alpine lakes. Other potential causes could be predation-inhibiting substances such as organophosphates, and herbicides used in agriculture, household and industrial detergents and surfactants, and fecal matter-associated compounds like indole, as well as antibiotic residues (Mitchell et al. 2020), all of which may be present in the ANK river water.

**Table 4.** α-diversity estimates (S=Richness, E=Evenness, H=Shannon index, D’= Simpson index) of Bdellovibrionaceae and communities of the Al-Nar/Kidron (ANK) river, and of the Al-Bireh (AB) WWTP data (italics) (Cohen et al. 2021). Bdellovibrionales and Bacteriovoracales diversity estimates between ANK and the AB fractions are significantly different, except for Bd Simpson index (t-test, *P* < 0.01, two-tailed).

	S	E	H	D`
**Bd**				
**ANK**	**35.2 ± 10.5**	**0.62 ± 0.06**	**2.19 ± 0.2**	**0.82 ± 0.04**
**AB F**	*69.7 ± 6.9*	*0.57 ± 0.09*	*2.41 ± 0.4*	*0.82 ± 0.1*
**AB L**	*64.4 ± 10.3*	*0.59 ± 0.06*	*2.44 ± 0.3*	*0.85 ± 0.06*
**Bx**				
**ANK**	**15.1 ± 11.2**	**0.25 ± 0.05**	**0.65 ± 0.3**	**0.36 ± 0.2**
**AB F**	*49.1 ± 9.7*	*0.31 ± 0.07*	*1.21 ± 0.3*	*0.51 ± 0.1*
**AB L**	*40.4 ± 6.3*	*0.30 ± 0.1*	*1.10 ± 0.4*	*0.45 ± 0.2*

**Figure 3.**
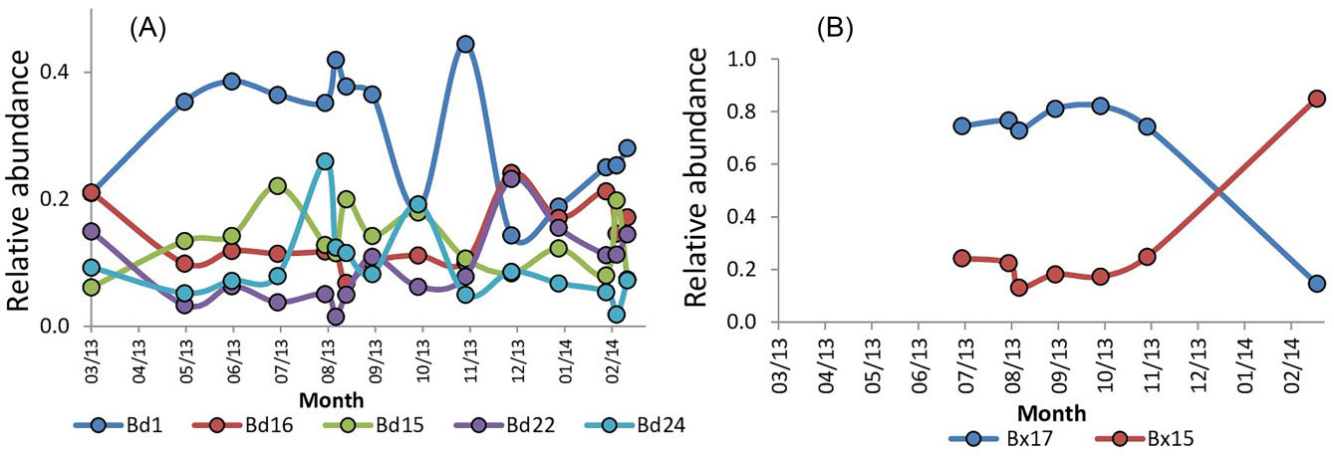
Annual dynamics of the most abundant Bdellovibrionaceae (Bd) (A) and Bacteriovoracaceae (Bx) (B) OTUs throughout the year-long time series in the ANK stream.

Temperature exerted a strong effect upon ANK's Bdellovibrionaceae populations, and, to a lesser extent, nitrate (Table S5). Among the dominant Bd OTUs (>0.1% in >90% of the samples), Bd1 was found year-long at high abundance with a population peak during the summer months; by contrast, populations of Bd16 and Bd22 were enhanced during the colder period. OTU Bd30 was mostly stable. Yet, summer-promoted or winter-promoted OTUs could exhibit large fluctuations (Fig. 3A). The temperature dependence of BALO communities has been noted numerous times (Staples and Fry 1973, Williams 1988, Paix et al. 2019, Yan et al. 2022), but its impact on the predator vs. indirect effects due to environmental changes (e.g. prey community structure, water composition) needs to be evaluated. Other dominant Bd OTUs shifted on a shorter time-frame (e.g. Bd1, Bd22, Bd24). A possible cause may be regulation by prey availability (Chen et al. 2011) or by Kill the Winner dynamics (Shapiro et al. 2009, Winter et al. 2010, Cohen et al. 2021). It can be noted that, respectively, Bd1 and Bd16 were found in all, and almost all core BALO communities in the floc and liquor fractions of three WWTPs (data from Cohen et al. 2021), suggesting that these populations are not geographically restricted, which may be due to an ability to sustain rather different environmental conditions. Phylogenetic analysis showed that very few of the Bd OTUs were related to the “classical” Bd strain *B. bacteriovorus* H-D100, which was isolated from soil (Stolp and Starr 1963). One OTU was closely related to the soil isolate *B. reynosensis* (Ajao et al. 2023), and three other Bd OTUs were in sister clades to known isolated representatives (Fig. S4). By contrast, only two Bx OTUs dominated, showing inverted patterns of abundance between summer and winter (Fig. 3B). Yet, we carefully suggest that other parameters, not included in the ones measured in the current study, may prevent the establishment of Bx populations.

## Conclusion

In an intermittent Mediterranean stream where WWTP effluent (e.g. with largely reduced organic loads compared with those found in the ANK river) could constitute up to 100% of the streamflow during the summer period, the river's microbial communities were rapidly restored within 1 km (Pascual-Benito et al. 2020). By comparison, the sampled stretch of the ANK river that remains severely polluted extends for more than 10 km and is situated more than 15 km away from the main source of sewage.

Our data, showing that the ANK river's microbiota are deficient in OM degraders and in nitrifiers, point at a largely reduced potential for water self-purification. In a recent study, Shang et al. (2021), using alternative stable state theory, found that, in addition to higher diversity, co-occurrence patterns of microbial communities in favorable (less impacted) river environments included Myxococcales and NItrospirales as key taxa, with Rhizobiales also forming large hubs. Our data show large fluctuations in community structure between sampling times, low evenness and low diversity, which indicate low overall stability; they also reveal a scarceness of BALO and Myxococcales predators, an absence or very low presence of Rhizobiales, Bacterioidetes and Planctomycetes, all of which are not only important degraders of organic compounds, but also large components in BALO co-occurrence networks in WWTPs and in freshwater (Feng et al. 2017, Ezzedine et al. 2020, Cohen et al. 2021). We thus further propose that the dearth of both facultative and obligate bacterial predators, along with the specific composition of the predatory micro-eukaryote community present in the stream, hamper the establishment of efficient trophic networks that help stabilize communities (Welsh et al. 2015, Cohen et al. 2021, Hungate et al. 2021, Lee et al. 2021, Petters et al. 2021) and contribute to increased community diversity (Winter et al. 2010). We propose that future studies on water self-purification of highly sewage-impacted streams may benefit from accentuated research on the role of micro-predators in re-establishing functional, water-purifying assemblages (top-down) vs. water compositional and environmental parameters and their effects on microbial degraders and recyclers (bottom-up). As for the ANK river, fortunately a restoration project has been initiated, and the river's water and riverine habitats are expected, if not to recover, then at least to significantly improve in future years (HaGihon 2023).

## Supplementary Material

fiae069_Supplemental_Files
