# Supplementary material for: Unbalanced predatory communities and a lack of microbial degraders characterize the microbiota of a highly sewage-polluted Eastern-Mediterranean stream
Source: FEMS Microbiol Ecol. 2024 Apr 29;100(6):fiae069. doi: 10.1093/femsec/fiae069 (PMC11099661; doi:10.1093/femsec/fiae069)
Supplement: fiae069_Supplemental_Files [file fiae069_supplemental_files.zip › Figures_Supplementary data_Final_submitted.pptx]

## Slide 1
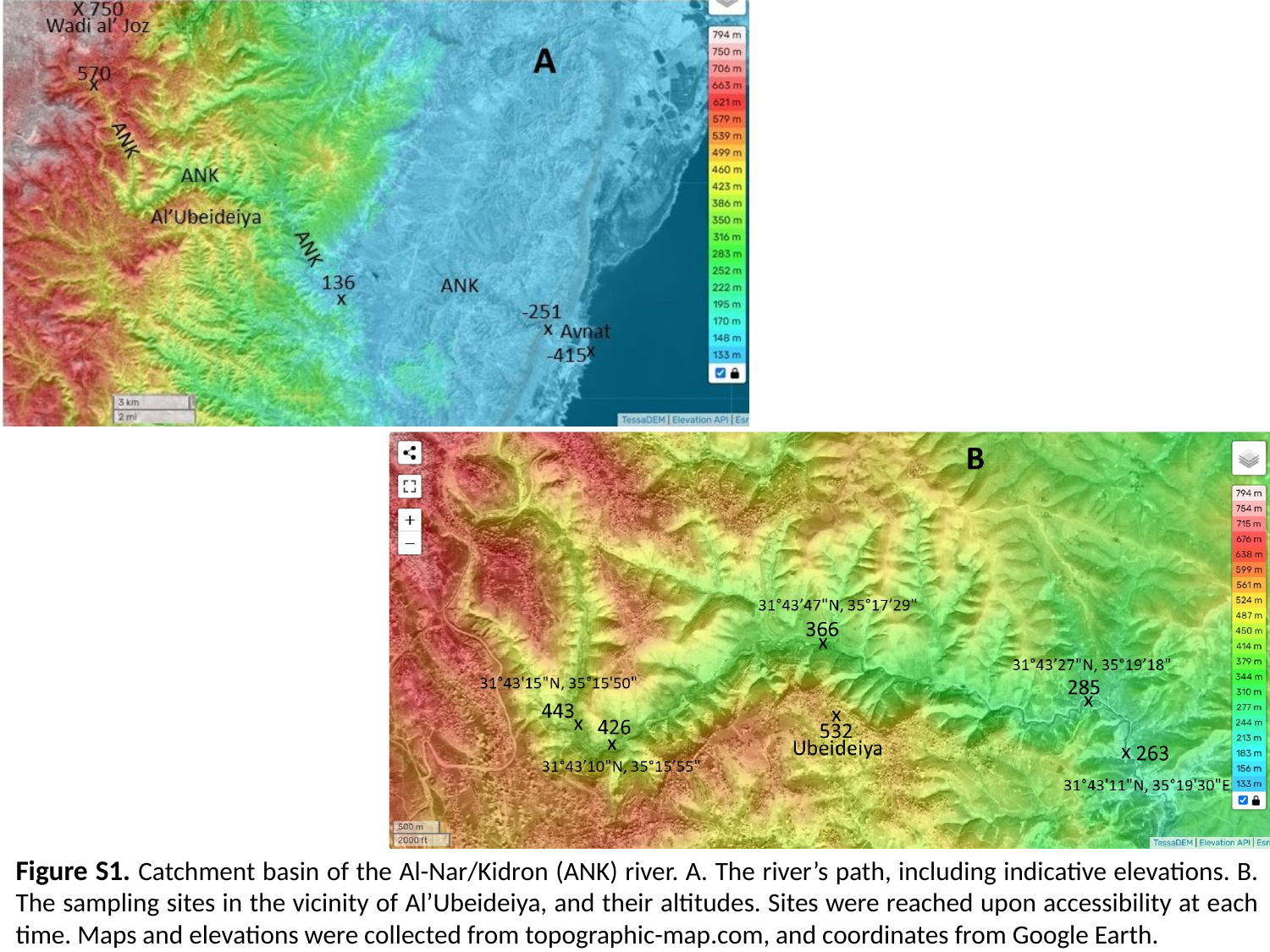

Figure S1. Catchment basin of the Al-Nar/Kidron (ANK) river. A. The river’s path, including indicative elevations. B. The sampling sites in the vicinity of Al’Ubeideiya, and their altitudes. Sites were reached upon accessibility at each time. Maps and elevations were collected from topographic-map.com, and coordinates from Google Earth.

## Slide 2
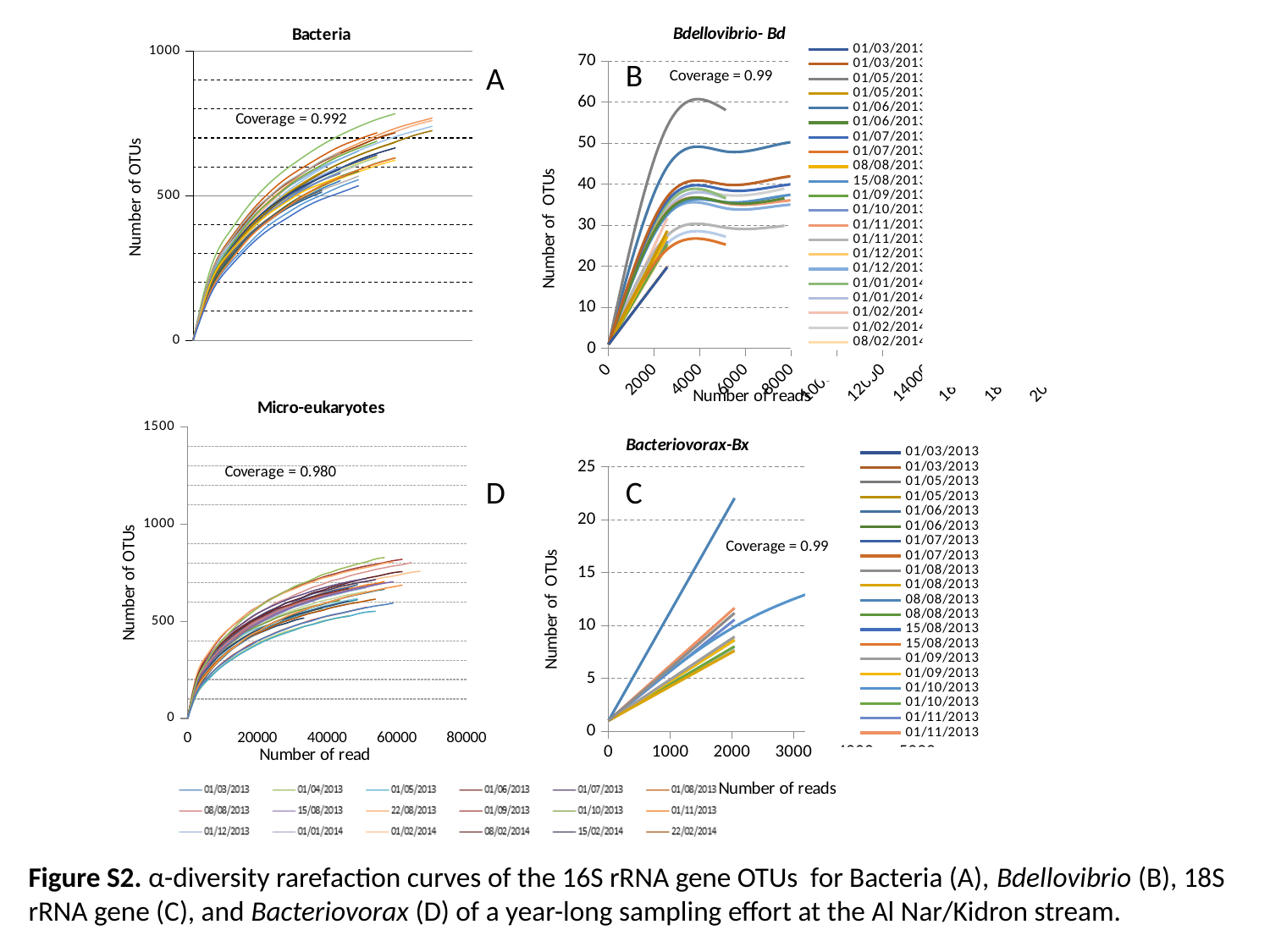

### Chart: Bdellovibrio- Bd
| Category | 01/03/2013 | 01/03/2013 | 01/05/2013 | 01/05/2013 | 01/06/2013 | 01/06/2013 | 01/07/2013 | 01/07/2013 | 08/08/2013 | 15/08/2013 | 01/09/2013 | 01/10/2013 | 01/11/2013 | 01/11/2013 | 01/12/2013 | 01/12/2013 | 01/01/2014 | 01/01/2014 | 01/02/2014 | 01/02/2014 | 08/02/2014 | 15/02/2014 |
|---|---|---|---|---|---|---|---|---|---|---|---|---|---|---|---|---|---|---|---|---|---|---|
### Chart: Bacteria
| Category | 01/03/2013 | 01/03/2013 | 01/05/2013 | 01/05/2013 | 01/06/2013 | 01/06/2013 | 01/07/2013 | 01/07/2013 | 01/08/2013 | 01/08/2013 | 08/08/2013 | 08/08/2013 | 15/08/2013 | 15/08/2013 | 01/09/2013 | 01/09/2013 | 01/10/2013 | 01/10/2013 | 01/11/2013 | 01/11/2013 | 01/12/2013 | 01/12/2013 | 01/01/2014 | 01/01/2014 | 01/02/2014 | 01/02/2014 | 08/02/2014 | 15/02/2014 | 15/02/2014 | 22/02/2014 | 22/02/2014 |
|---|---|---|---|---|---|---|---|---|---|---|---|---|---|---|---|---|---|---|---|---|---|---|---|---|---|---|---|---|---|---|---|
B
A
Coverage = 0.99
Number of OTUs
### Chart: Micro-eukaryotes
| Category | 01/03/2013 | 01/03/2013 | 01/05/2013 | 01/05/2013 | 01/06/2013 | 01/06/2013 | 01/07/2013 | 01/07/2013 | 01/08/2013 | 01/08/2013 | 08/08/2013 | 08/08/2013 | 15/08/2013 | 15/08/2013 | 01/09/2013 | 01/09/2013 | 01/10/2013 | 01/10/2013 | 01/11/2013 | 01/11/2013 | 01/12/2013 | 01/12/2013 | 01/01/2014 | 01/01/2014 | 01/02/2014 | 01/02/2014 | 08/02/2014 | 15/02/2014 | 15/02/2014 | 22/02/2014 | 22/02/2014 |
|---|---|---|---|---|---|---|---|---|---|---|---|---|---|---|---|---|---|---|---|---|---|---|---|---|---|---|---|---|---|---|---|
### Chart: Bacteriovorax-Bx
| Category | 01/03/2013 | 01/03/2013 | 01/05/2013 | 01/05/2013 | 01/06/2013 | 01/06/2013 | 01/07/2013 | 01/07/2013 | 01/08/2013 | 01/08/2013 | 08/08/2013 | 08/08/2013 | 15/08/2013 | 15/08/2013 | 01/09/2013 | 01/09/2013 | 01/10/2013 | 01/10/2013 | 01/11/2013 | 01/11/2013 | 01/12/2013 | 01/12/2013 | 01/01/2014 | 01/01/2014 | 01/02/2014 | 01/02/2014 | 08/02/2014 | 15/02/2014 | 15/02/2014 | 22/02/2014 | 22/02/2014 |
|---|---|---|---|---|---|---|---|---|---|---|---|---|---|---|---|---|---|---|---|---|---|---|---|---|---|---|---|---|---|---|---|D
C
Coverage = 0.99
Figure S2. α-diversity rarefaction curves of the 16S rRNA gene OTUs for Bacteria (A), Bdellovibrio (B), 18S rRNA gene (C), and Bacteriovorax (D) of a year-long sampling effort at the Al Nar/Kidron stream.

## Slide 3
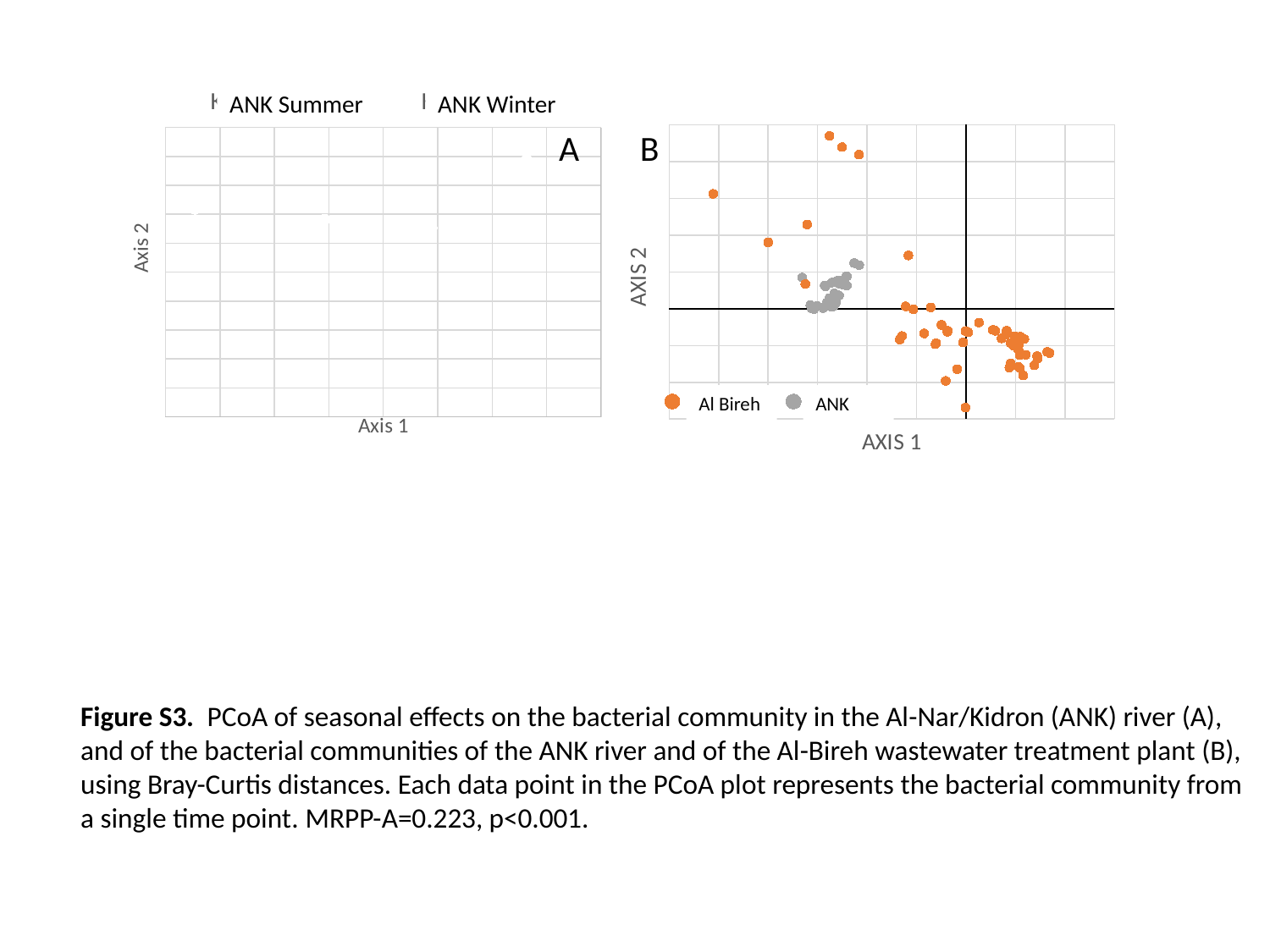

### Chart
| Category | | |
|---|---|---|ANK Summer
ANK Winter
### Chart
| Category | | |
|---|---|---|A
B
Al Bireh
ANK
Figure S3. PCoA of seasonal effects on the bacterial community in the Al-Nar/Kidron (ANK) river (A), and of the bacterial communities of the ANK river and of the Al-Bireh wastewater treatment plant (B), using Bray-Curtis distances. Each data point in the PCoA plot represents the bacterial community from a single time point. MRPP-A=0.223, p<0.001.

## Slide 4
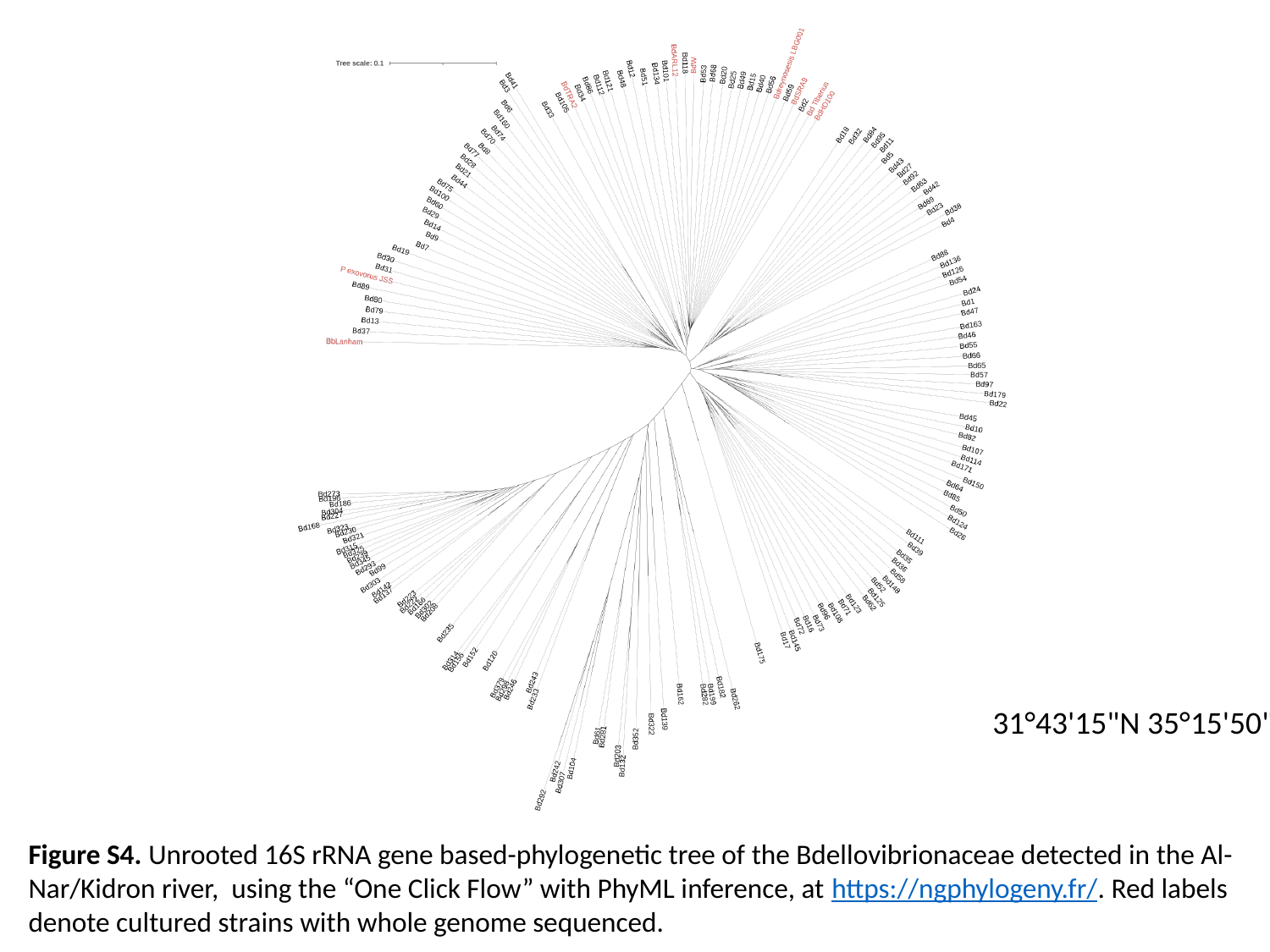

⁦31°43'15"N⁩ ⁦35°15'50"E⁩
Figure S4. Unrooted 16S rRNA gene based-phylogenetic tree of the Bdellovibrionaceae detected in the Al-Nar/Kidron river, using the “One Click Flow” with PhyML inference, at https://ngphylogeny.fr/. Red labels denote cultured strains with whole genome sequenced.
