# Supplementary material for: Unbalanced predatory communities and a lack of microbial degraders characterize the microbiota of a highly sewage-polluted Eastern-Mediterranean stream
Source: FEMS Microbiol Ecol. 2024 Apr 29;100(6):fiae069. doi: 10.1093/femsec/fiae069 (PMC11099661; doi:10.1093/femsec/fiae069)
Supplement: fiae069_Supplemental_Files [file fiae069_supplemental_files.zip › Supplementary data tables_Final.docx]

**Supplementary Tables**

**Table S1.** Alpha diversity parameters of the Bacteria (16S rRNA)(A) and of the micro-eukaryote (18S rRNA) communities (B) retrieved from publications on microbial communities analyses of river waters.

| **A**  **16S rRNA gene** | Ting et al., 2021 | Korajkic et al. 2015 | Wang et al., 2018 | Nakatsu et al., 2019 | Pascual-Benito et al., 2021 | Shang et al., 2021 |
| --- | --- | --- | --- | --- | --- | --- |
| **Shannon** | 4.3+2.7 | 3.95-6.34 | 6.97-7.89 | 6.6 | 5.5-6 | ~6.7-7.5 |
| **Richness (OTUs)** | 198+145 |  | 2331-3686 |  | 8062- 16,645* |  |

* Amplicon sequence variants

| **B**  **18S rRNA gene** | Li et al, 2018** | Abdullah Al et al., 2021 | Xu et al., 2020*** | Choi et al., 2022 | Muhammad et al., 2021 | Ting et al., 2021 |
| --- | --- | --- | --- | --- | --- | --- |
| **Shannon** | 3.73-3.82 | 3.16 ± 0.23 | 5.51, 3.54-~4.4 | 2.82-5.86 | 2.11-3.99 | 6.07+1.42 |
| **Simpson** | 0.896-0.912 | 0.88 ± 0.00 |  |  |  |  |
| **Evenness** |  | 0.62 | 0.56,  0.38-0.47 | 0.62 | 0.08-0.3 |  |
| **Richness (OTUs)** | 655-692 | 209 | 705, 396-475 | 59-238 | 90–174 | 438 |

* Amplicon sequence variants - “High pollution” site

**Low salinity sites (<1 ^0^/00)

***Low salinity, source, urban- and agriculture-impacted sites

**Table S2** Pearson correlations (|r|) between the bacterial and micro-eukaryote communities and environmental parameters in the Al-Nar/Kidron river (ANK). Temperature (^o^C); Salinity (ppm); conductivity (µS/m); Total suspended solids(TSS)(mg.l^-1^ ); Biological oxygen demand (BOD, (mg.l^-1^); Chemical oxygen demand (COD, (mg.l^-1^); Nitrate (NO_2_,ppm); Nitrate (NO_3_,ppm); Phosphorus (PO_4_,ppm); Total organic carbon (TOC, mg.l^-1^); Total dissolved solids (TDS, mg.l^-1^).

|  |  | **ANK** |
| --- | --- | --- |
| **16S rRNA gene** | **Temp [°C]** | 0.851 |
|  | **Salinity** | 0.585 |
|  | **pH** | 0.056 |
|  | **Conductivity** | 0.333 |
|  | **TSS** | -0.48 |
|  | **BOD** | -0.257 |
|  | **COD** | -0.196 |
|  | **NO2** | -0.146 |
|  | **NO3** | 0.578 |
|  | **NH4** | 0.227 |
|  | **PO4** | -0.089 |
|  | **TOC** | 0.338 |
|  | **TDS** | 0.274 |
| **18S rRNA gene** | **Temp [°C]** | 0.804 |
|  | **Salinity** | 0.588 |
|  | **pH** | 0.115 |
|  | **Conductivity** | 0.374 |
|  | **TSS** | -0.51 |
|  | **BOD** | -0.139 |
|  | **COD** | -0.189 |
|  | **NO2** | -0.114 |
|  | **NO3** | 0.486 |
|  | **NH4** | 0.221 |
|  | **PO4** | -0.065 |
|  | **TOC** | 0.418 |
|  | **TDS** | 0.298 |

**Table S3**. Characteristics of the Al-Nar/Kidron river (ANK) water samples of this study. Samples were taken over a year, on a monthly basis as averages and standard error from the mean. Temperature (^o^C); Salinity (ppm); conductivity (µS/m); Total suspended solids(TSS)(mg.l^-1^ ); Biological oxygen demand (BOD, (mg.l^-1^); Chemical oxygen demand (COD, (mg.l^-1^); Nitrate (NO_2_,ppm); Nitrate (NO_3_,ppm); Phosphorus (PO_4_,ppm); Total organic carbon (TOC, mg.l^-1^); Total dissolved solids (TDS, mg.l^-1^).

| **Temp** | 23.9+7.2 |
| --- | --- |
| **Salinity** | 0.625+0.1 |
| **pH** | 7.28+0.3 |
| **Conductivity** | 1541.38+128.8 |
| **TSS** | 222.8+31.4 |
| **COD** | 509+294.6 |
| **BOD** | 286+104.3 |
| **NO_2_** | 0.129+0.2 |
| **NO_3_** | 44.38+24.6 |
| **NH_4_** | 65.2+23.1 |
| **PO_4_** | 6.6+3.5 |
| **TOC** | 72.1+21.7 |
| **TDS** | 854+74 |

**Table S4.** Maximal relative abundance (R.A., annual average and standard error, in % of all OTUs, and in parenthesis, of identified OTUs) of potential micro-eukaryote bacterial predators.

| **Phylum** | **R.A.%** |
| --- | --- |
| SAR, Ciliophora, 102 OTUs | 3.86+0.012 (17.2) |
| Excavata, 35 OTUs | 3.31+0.04 (14.8) |
| Amoebozoa, 28 OTUs | 0.75+0.006 (3.35) |

**Table S5.** Pearson correlations (|r|) between the Bdellovibrionaceae and the Bacteriovoracaeae communities and environmental parameters in the Al-Nar/Kidron river (ANK). Temperature (^o^C); Salinity (ppm); conductivity (µS/m); Total suspended solids(TSS)(mg.l^-1^ ); Biological oxygen demand (BOD, (mg.l^-1^); Chemical oxygen demand (COD, (mg.l^-1^); Nitrate (NO_2_,ppm); Nitrate (NO_3_,ppm); Phosphorus (PO_4_,ppm); Total organic carbon (TOC, mg.l^-1^); Total dissolved solids (TDS, mg.l^-1^).

|  |  | **ANK** |
| --- | --- | --- |
| **Bdellovibrionaceae** | **Temp** | 0.83 |
|  | **Salinity** | 0.399 |
|  | **pH** | 0.011 |
|  | **Conductivity** | 0.229 |
|  | **TSS** | 0.616 |
|  | **BOD** | 0.106 |
|  | **COD** | 0.312 |
|  | **NO2** | 0.034 |
|  | **NO3** | 0.588 |
|  | **NH4** | 0.033 |
|  | **PO4** | 0.018 |
|  | **TOC** | 0.198 |
|  | **TDS** | 0.383 |
| **Bacteriovoracaceae** | **Temp** | 0.664 |
|  | **Salinity** | 0.168 |
|  | **pH** | 0.337 |
|  | **Conductivity** | 0.354 |
|  | **TSS** | 0.411 |
|  | **BOD** | 0.877 |
|  | **COD** | 0.68 |
|  | **NO2** | 0.358 |
|  | **NO3** | 0.521 |
|  | **NH4** | 0.068 |
|  | **PO4** | 0.851 |
|  | **TOC** | 0.258 |
|  | **TDS** | 0.584 |

**References**

Abdullah Al, M., Xue, Y., Xiao, P., Chen, H., Zhang, C., Duan, M., and Yang, J. (2021) DNA metabarcoding reveals the significant influence of anthropogenic effects on microeukaryotic communities in urban waterbodies. *Environmental Pollution* **285**: 117336.

Choi, T.-J., Malik, A., An, H.-E., Kim, J.-I., Dinh Do, T., and Kim, C.-B. (2022) Seasonal Diversity of Microeukaryotes in the Han River, Korea Through 18S rRNA Gene Metabarcoding. *Evolutionary Bioinformatics* **18**: 11769343221074688.

Li, R., Jiao, N., Warren, A., and Xu, D. (2018) Changes in community structure of active protistan assemblages from the lower Pearl River to coastal Waters of the South China Sea. *European Journal of Protistology* **63**: 72–82.

Korajkic, A., Parfrey, L.W., McMinn, B.R., Baeza, Y.V., VanTeuren, W., Knight, R., and Shanks, O.C. (2015) Changes in bacterial and eukaryotic communities during sewage decomposition in Mississippi river water. *Water Research* **69**: 30–39.

Muhammad, B.L., Kim, T., and Ki, J.-S. (2021) 18S rRNA Analysis Reveals High Diversity of Phytoplankton with Emphasis on a Naked Dinoflagellate Gymnodinium sp. at the Han River (Korea). *Diversity* **13**:.

Nakatsu, C.H., Byappanahalli, M.N., and Nevers, M.B. (2019) Bacterial Community 16S rRNA Gene Sequencing Characterizes Riverine Microbial Impact on Lake Michigan. *Frontiers in Microbiology* **10**:.

Pascual-Benito, M., Ballesté, E., Monleón-Getino, T., Urmeneta, J., Blanch, A.R., García-Aljaro, C., and Lucena, F. (2020) Impact of treated sewage effluent on the bacterial community composition in an intermittent mediterranean stream. *Environmental Pollution* **266**: 115254.

Shang, J., Zhang, W., Chen, X., Li, Y., Niu, L., Wang, L., and Zhang, H. (2021) How environmental stress leads to alternative microbiota states in a river ecosystem: A new insight into river restoration. *Water Research* **203**: 117538.

Ting, A.S.Y., Zoqratt, M.Z.H.M., Tan, H.S., Hermawan, A.A., Talei, A., and Khu, S.T. (2021) Bacterial and eukaryotic microbial communities in urban water systems profiled via Illumina MiSeq platform. *3 Biotech* **11**: 40.

Wang, L., Zhang, J., Li, H., Yang, H., Peng, C., Peng, Z., and Lu, L. (2018) Shift in the microbial community composition of surface water and sediment along an urban river. *Science of the Total Environment* **627**: 600–612.

Xu, H., Zhang, S., Ma, G., Zhang, Y., Li, Y., and Pei, H. (2020) 18S rRNA gene sequencing reveals significant influence of anthropogenic effects on microeukaryote diversity and composition along a river-to-estuary gradient ecosystem. *Science of The Total Environment* **705**: 135910.
