# Supplementary material for: Unbalanced predatory communities and a lack of microbial degraders characterize the microbiota of a highly sewage-polluted Eastern-Mediterranean stream
Source: FEMS Microbiol Ecol. 2024 Apr 29;100(6):fiae069. doi: 10.1093/femsec/fiae069 (PMC11099661; doi:10.1093/femsec/fiae069)
Supplement: fiae069_Supplemental_Files [file fiae069_supplemental_files.zip › Supplementary data_materials_and_methods_information.docx]

**Al Nar/Kidron (ANK) river geography and climate**. The ANK river is a seasonal, winter stream. with its head in Wadi Joz in Jerusalem (31o47’20”N, 35 o14’16”E, elevation 750 m). It flows eastward on a 34 km-long course, mostly over chalk rocks, and passes through a series of canyons to reach the shores of the Dead Sea at Avnat (31o39’56’’N, 35o27’00’’; -415 m)(Figure S1A). Rainfall data (average annual precipitation; % of precipitation between December and March) for the head of the river, the sampled stretch of the river, and the mouth of the river (at Ein Gedi, ,31o27’06’’, 35o23’53’’, elevation -415 m, 25 km south of Avnat, also below the cliffs of the Judean mountains along the Dead Sea shore; as no rainfall data are available for Avnat) were as follows: At Jerusalem, 510 mm; >83%; at Al’ Ubeideiya, about 17 km on the river course east of central Jerusalem (31o43’26’’N, 35o17’30’E’, elevation 532 m), 262.0 mm and 280.8 mm in 2012/2013 and 2013/2014, respectively; >85% (Safi and Mohammad 2019); At Ein Gedi, 48.9 mm; >74%. Data for Jerusalem and Ein Gedi were collected from Israel Meteorological Service (IMS 2023, in Hebrew). In addition to winter rains, the ANK river receives 11 million m3 of raw, untreated sewage annually, contributed by 200,000 inhabitants living within the 115 km2 of the river’s drainage basin, mostly in eastern Jerusalem, Al’ Ubeideiya (pop. circa 15,000) and other smaller communities along the river’s course.

***Bacterial quantification by real-time qPCR***. qPCR was performed as in Cohen et al. (2021). Standards were prepared by inserting a 1467-bp fragment of the *Bdellovibrio bacteriovorus* HD100 *and Bacteriovorax stolpii* UKi2 strain 16S rRNA gene amplified with primers 27F and 1492R respectively into a PGEM-T easy plasmid vector system (Promega, WI, USA). Ten-times serial dilutions from 10^3^ to 10^10^ plasmid copies per reaction were used to construct standard qPCR curves and plasmid copy numbers calculated. For total bacteria, primer pair 1048F-1175R was used to quantify the 16S rRNA gene copy number. Each 25μl reaction consisted of 12.5μl of SYBR® Green PCR Master Mix (Applied Biosystems, USA) 1μl of each primer, 1μl of DNA and 9.5μl of PCR grade DDW. Thermal cycling was performed as follows: 50°C (2 min) and 95°C (10 min), 40 cycles of 95°C (15 sec), 60°C (1 min), followed by dissociation; Melt curve was used for all experiments from 55°C to 95°C. For *Bdellovibrio* 16S rRNA gene quantification primers Bd347F-Bd549R were used in a reaction mix and thermal cycling conditions as above except for the use of 45 cycles. For *Bacteriovorax* 16S rRNA gene quantification primers BacF519 - BacR677^98^ were used in a reaction mix as above. Thermal cycling conditions were: 50°C (2 min), 94°C (2 min), 45 cycles at 94°C (30 sed), 62°C (10 sec) and 72°C (10 sec), followed by dissociation; Melt curve was used for all experiments from 55°C to 95°C. Reactions were performed in a 96-well plate (Applied Biosystems) with MicroAmp® Optical Adhesive Film (Applied Biosystems, USA) in a Step One plus Real-time PCR System (Applied Biosystems). Total QPCR counts (16S rRNA bacterial genes ml^-1^) were estimated based on a standard curve with 100% efficiency factoring in the cycle threshold value of the particular sample obtained, considering that 25 mL of sample was used for DNA extraction, DNA was eluted in 50μl of DDW and 1μl of this DNA was 1- fold diluted and used in the QPCR reaction.

***Microbial diversity and statistical analyses***. As in Cohen et al. (2019, 2021). OTUs were arranged in a data matrix where each row was a single sample and each column a specific OTU; each data point in the matrix represented the abundance of the particular OTU in a particular sample, relativized to the sampling effort (i.e. the number of MiSeq reads obtained from that sample). Rarefaction curves were calculated using CLC-Bio genomics workbench 8.5 (Qiagen, Aarhus, Denmark). Read abundance data were not rarefied. α-Diversity parameters (Shannon, Simpson, Richness and Evenness) and Multivariate analysis were performed in PC-ORD v6.0 (MjM Software, Gleneden Beach, OR) with Sorensen (Bray-Curtis) dissimilarities. Ordinations were performed with PCoA (Principal Coordinates Analysis, a geometric technique that converts a matrix of distances between points in multivariate space into a projection that maximizes the amount of variation along a series of orthogonal axes) at 500 iterations. Differences between sample groups were calculated with the multi-response permutation procedure (MRPP). The size of the difference between groups was represented by the A-statistic of the MRPP test (low values indicate higher similarity between samples), while its significance was identified by the MRPP P-value. Correlations between microbial communities (as represented by the PCoA 1^st^ axis) and environmental parameters were calculated by Pearson’s correlation coefficient.

***16S rRNA and 18S rRNA gene community sequencing and analysis***. This analysis was performed as in Cohen et al. (2019, 2021). MiSeq Illumina sequencing (Carlsbad, CA, USA) was performed using bacterial 16S rRNA primers 515F (5’GTGCCAGCMGCCGCGGTAA-3’) and 806R (5’- GGACTACHVGGGTWTCTAAT-3’) targeting the V4 region, Bdellovibrionales primers Bd824F (‘5-ACTTGTTGTTGGAGGTAT-3’) and Bd1222R (‘5-TTGTAGCACGTGTGTAG-‘3), Bacteriovoracales-primers Bx341F (5`-CTACGGGAGGCAGCAG-3’) and Bx672RC (5’-TACCCCTACATGCGAAATTCC-3’), and eukaryotic 18S rRNA primers Euk_1391 (5’- GTACACACCGCCCGTC-3’) and Euk_Br (5’-TGATCCTTCTGCAGGTTCACCTAC-3’) targeting the V9 region. Sequences were processed using CLC-Bio genomics Workbench 8.5 (Qiagen, Aarhus, Denmark). Forward and reverse 16S rRNA reads were merged whereas only the forward 18S rRNA reads were considered for analysis. All reads were screened for quality and trimmed as outlined in the CLC-Bio MiSeq pipeline, aligned to the SILVA reference alignment database, and filtered so that they all overlap (with no overhang). Finally, chimeric reads were removed using the CLC-Bio proprietary algorithm tool. Pairwise distances were calculated between all DNA reads, and reads were subsequently clustered into operational taxonomic units (OTUs) at 0.03 divergence (>97% similarity). OTU affiliation was determined based on SILVA taxonomy.
